# Cross-protective immunity against influenza A/H1N1 virus challenge in mice immunized with recombinant vaccine expressing HA gene of influenza A/H5N1 virus

**DOI:** 10.1186/1743-422X-10-291

**Published:** 2013-09-22

**Authors:** Song Yang, Shumeng Niu, Zhihua Guo, Ye Yuan, Kun Xue, Sinan Liu, Hong Jin

**Affiliations:** 1 Department of Pathogen Biology, China Medical University, Shenyang, Liaoning, PR China; 2 Department of animal husbandry and veterinary, Liao Ning Medical University, Jin Zhou, Liaoning, PR China

**Keywords:** Influenza virus, Heterosubtypic immunity, Vaccine, Cellular immune response, Cytotoxic T-lymphocytes

## Abstract

**Background:**

Influenza virus undergoes constant antigenic evolution, and therefore influenza vaccines must be reformulated each year. Time is necessary to produce a vaccine that is antigenically matched to a pandemic strain. A goal of many research works is to produce universal vaccines that can induce protective immunity to influenza A viruses of various subtypes. Despite intensive studies, the precise mechanisms of heterosubtypic immunity (HSI) remain ambiguous.

**Method:**

In this study, mice were vaccinated with recombinant virus vaccine (rL H5), in which the hemagglutinin (HA) gene of influenza A/H5N1 virus was inserted into the LaSota Newcastle disease virus (NDV) vaccine strain. Following a challenge with influenza A/H1N1 virus, survival rates and lung index of mice were observed. The antibodies to influenza virus were detected using hemagglutination inhibition (HI). The lung viral loads, lung cytokine levels and the percentages of both IFN-γ^+^CD4^+^ and IFN-γ^+^CD8^+^ T cells in spleen were detected using real-time RT-PCR, ELISA and flow cytometry respectively.

**Results:**

In comparison with the group of mice given phosphate-buffered saline (PBS), the mice vaccinated with rL H5 showed reductions in lung index and viral replication in the lungs after a challenge with influenza A/H1N1 virus. The antibody titer in group 3 (H1N1-H1N1) was significantly higher than that in other groups which only low levels of antibody were detected. IFN-γ levels increased in both group 1 (rL H5-H1N1) and group 2 (rL H5 + IL-2-H1N1). And the IFN-γ level of group 2 was significantly higher than that of group 1. The percentages of both IFN-γ^+^CD4^+^ and IFN-γ^+^CD8^+^ T cells in group 1 (rL H5-H1N1) and group 2 (rL H5 + IL-2-H1N1) increased significantly, as measured by flow cytometry.

**Conclusion:**

After the mice were vaccinated with rL H5, cross-protective immune response was induced, which was against heterosubtypic influenza A/H1N1 virus. To some extent, cross-protective immune response can be enhanced by IL-2 as an adjuvant. Cellular immune responses may play an important role in HSI against influenza virus.

## Introduction

Influenza A virus (IAV), a member of the *Orthomyxoviridae* family, possesses a negative strand RNA genome made up of eight gene segments. Based on surface proteins hemagglutinin (HA) and neuraminidase (NA), IAV are classified 17 HA and 10 NA subtypes [1,2]. Influenza virus undergoes rapid antigenic evolution by accumulation of mutations and through genetic reassortments of segments.

IAV causes frequent epidemics, occasional pandemics and yearly seasonal outbreaks in humans, resulting in significant morbidity and mortality worldwide [3-5]. Vaccination has been one of the most effective means of protection against IAV. Due to the constant antigenic evolution of IAV, influenza vaccines must be reformulated each year. Although these vaccines are efficacious, they arrive late and after the peak of a pandemic. Therefore, we wish to investigate universal vaccines that can induce protective immunity to IAV of various subtypes [6]. Heterosubtypic immunity (HSI) is the basis of creating universal influenza vaccines. Evidence supports that cytokines and T cell responses play crucial roles in HSI to influenza virus [7,8]. Despite intensive studies, the precise mechanisms of HSI remain ambiguous.

In this study, mice were vaccinated with recombinant virus vaccine (rL H5), in which the HA gene of influenza A/H5N1 virus was inserted into LaSota Newcastle disease virus (NDV) vaccine strain. The mice were then challenged with influenza A/H1N1 virus. Furthermore, we co-administered intraperitoneally recombinant murine interleukin-2 (rIL-2) with the rL H5 to enhance HSI. We found that vaccination with rL H5 provided cross-protection against a lethal challenge with an antigenically distinct influenza A/H1N1 virus and produced significant changes in the levels of some cytokines and the percentages of both IFN-γ^+^CD4^+^ and IFN-γ^+^CD8^+^ T cells in lung and spleen. We also found that rIL-2 co-administered with the rL H5 could increase the survival rate of mice, reduce viral replication in lung and improve the IFN-γ production.

## Results

### Clinical outcome and lung index following influenza A/H1N1 virus infection

To determine whether primary immunization with the rL H5 vaccine can protect against a subsequent infection with heterosubtypic influenza virus, female 6–8 weeks-old C57BL/6 mice were immunized with the rL H5 vaccine and infected with influenza A/H1N1 virus with or without IL-2. The mice were monitored for body weight loss and survival rates.

**Figure 1:**
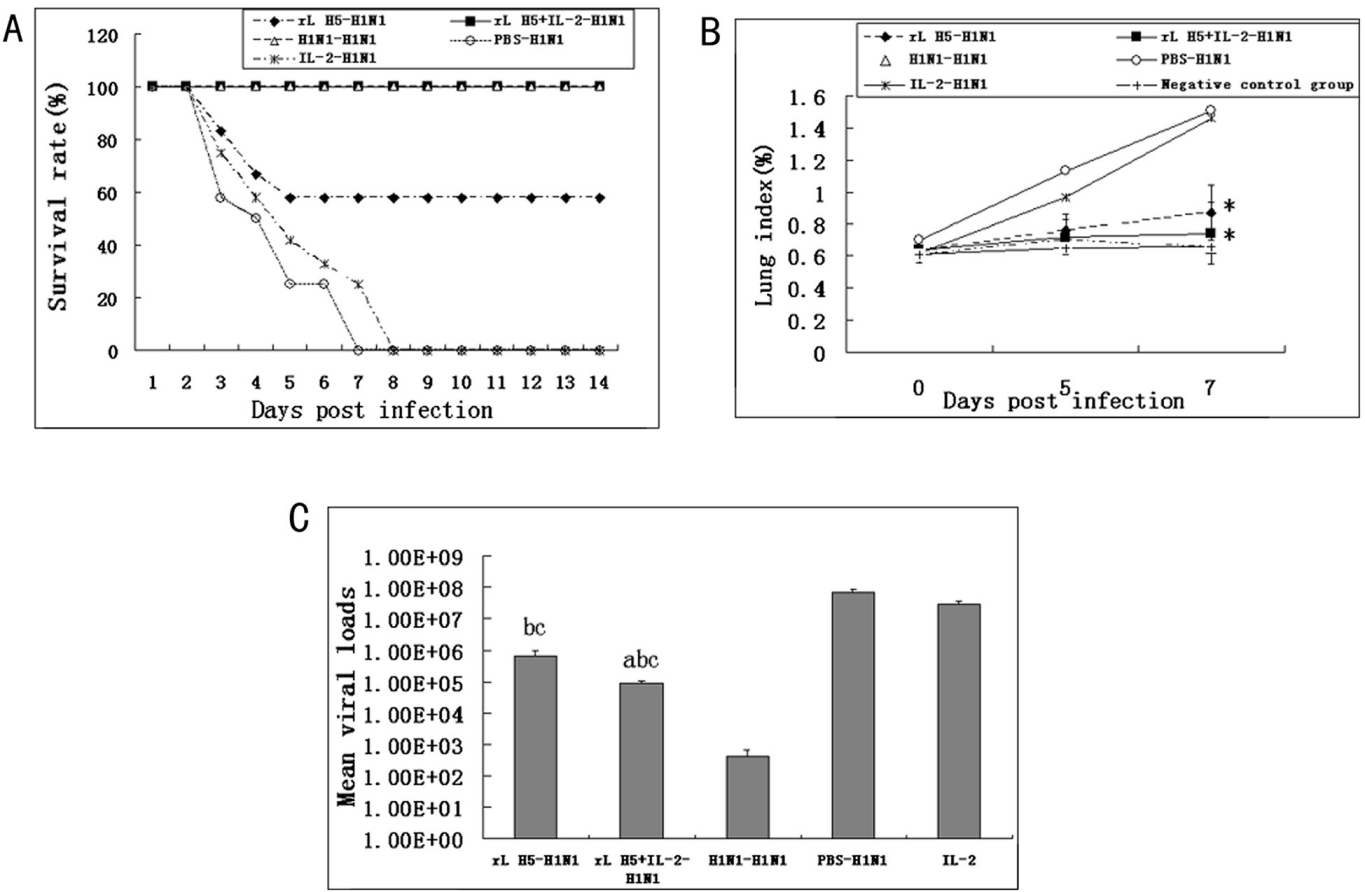
**Clinical outcome and lung index following influenza A/H1N1 virus infection. (A)** Survival rate of mice after infection with influenza A/H1N1 virus, which was significantly higher after day 6 for the rL H5-primed mice. n = 12/group. **(B)** The lung index of mice after infection with influenza A/H1N1 virus (*, as compare with group 4 [PBS-H1N1], P < 0.05). n = 3/group. **(C)** Lung viral loads on day 5 p.i. with influenza A/H1N1 virus (**a**, as compare with group 1 [rL H5-H1N1], P < 0.05; **b**, as compare with group 3 [H1N1- H1N1], P < 0.05; **c**, as compare with group 4 [PBS-H1N1], P < 0.05). n = 3/group.

The mice in group 2 (rL H5 + IL-2-H1N1) and group 3 (H1N1-H1N1) all survived after infection. The survival rate of mice in group 1 (rL H5-H1N1) was 58% at day 14 post-infection (p.i.). The mice in group 4 (PBS-H1N1) were all dead at day 7 p.i. (Figure 1A). The survival rate of group 2 (rL H5 + IL-2-H1N1) was higher than the survival rate of group 1 (rL H5-H1N1). Mice vaccinated with NDV were all dead at day 7 p.i. (data not shown). These results demonstrated that there was cross-protection between the H5 and H1 subtypes of influenza virus and that IL-2 as adjuvant could increase the survival rate of mice. NDV and IAV had not cross-protection.

The low lung index correlated well with strong protection during IAV infection. The lung index of group 1 (rL H5-H1N1) was 0.87%, group 2 (rL H5 + IL-2-H1N1) was 0.74%, and group 4 (PBS-H1N1) was 1.51% at day 7 p.i.. The lung indexes of group 1 and group 2 were lower than in group 4 at day 7 p.i. (P < 0.05) (Figure 1B). These data showed that rL H5 vaccine could prevent the mice from weights loss and inflammation of lung when they were challenged with influenza A/H1N1 virus.

### Viral load in the lungs of mice

The viral loads in the lungs of the mice were assessed using a real-time RT-PCR method at day 5 p.i. (Figure 1C). The lung viral loads of group 1 (rL H5-H1N1) and group 2 (rL H5 + IL-2-H1N1) were significantly lower than group 4 (P < 0.05). The lung viral loads of group 2 were lower than group 1 (P < 0.05). These data demonstrated that rL H5 induced cross-protection against A/H1N1 virus infection which may be enhanced by IL-2.

### Hemagglutination inhibition antibody responses

To determine whether the antibody had the protective effect against influenza A/H1N1 virus, we detected the levels of antibodies against influenza A/H1N1 virus in sera of mice by Hemagglutination inhibition (HI) assay (Table 1). The antibody titer in group 3 (H1N1-H1N1) was 640 at day 14 p.i.. It was significantly higher compared with the other groups that only low levels of antibody were detected. These data demonstrated that the levels of antibody might play a critical role in homologous influenza virus infection, but showed a negative correlation between HSI and antibody levels.

**Table 1:** Antibody titers in sera of mice infected with H1 influenza virus

**Experimental group**	**Antibody titers against H1 virus (HI)**	
	**Day 7**	**Day 14**
**rLH5-H1N1**	**<40**	**<40**
**rLH5 + IL-2-H1N1**	**<40**	**<40**
**H1N1-H1N1**	**320**	**640**
**PBS-H1N1**	**n.d.**	**n.d.**
**IL-2-H1N1**	**<40**	**n.d.**

A titer of <40 represents the lower limit of detection.

n.d. = not done. Animals did not survive until these time points.

### Cytokine expression in the lungs of mice

**Figure 2:**
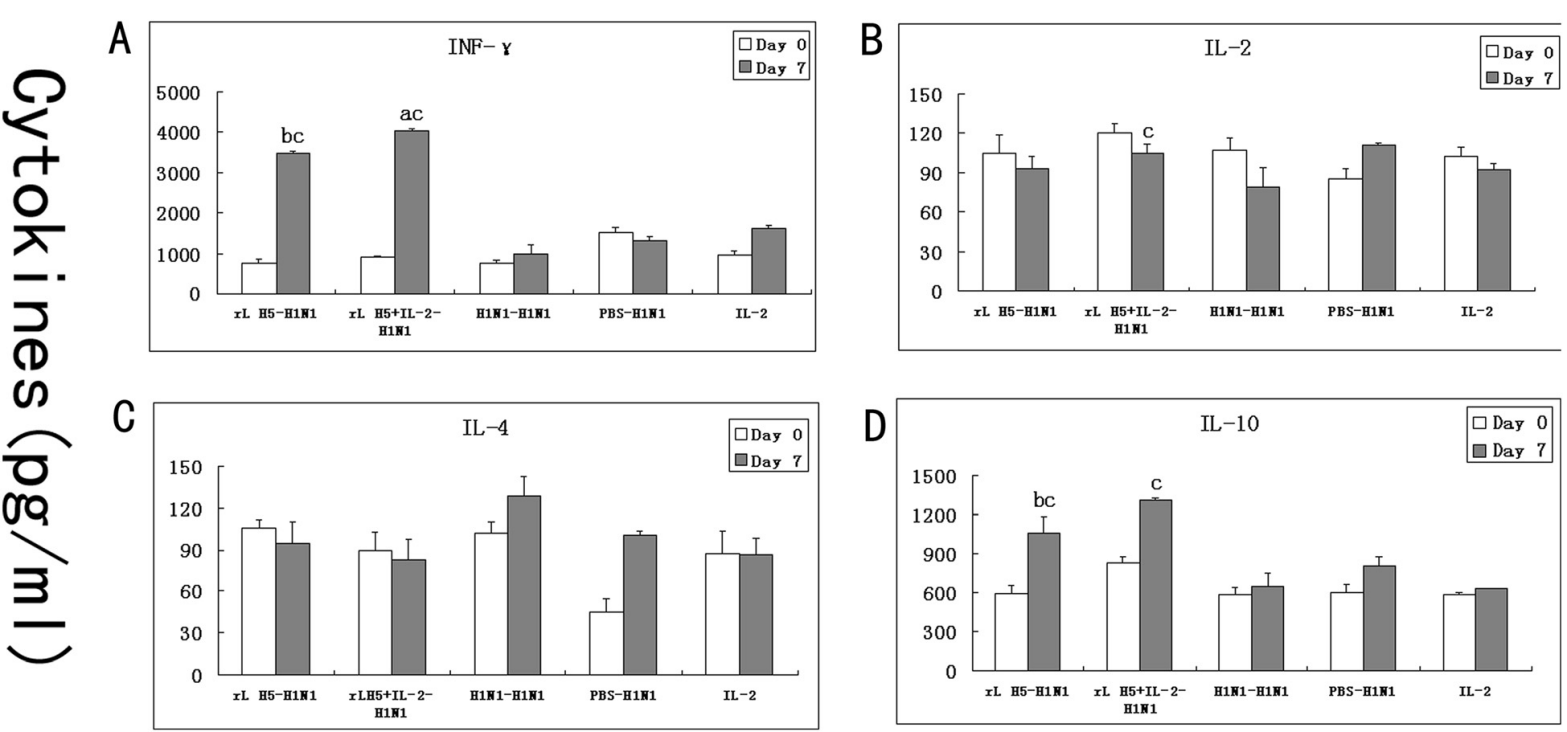
**Induction of cytokines by influenza A/H1N1 virus.** The levels of IFN-γ **(A)**, IL-2 **(B)**, IL-4 **(C)** and IL-10 **(D)** in the lung suspensions were detected by ELISA assay (**a**, as compare with group 1 (rL H5-H1N1), P < 0.05; **b**, as compare with group 3 (H1N1- H1N1), P < 0.05; **c**, as compare with 0day of the same group, P < 0.05). n = 3/group.

Lung suspensions were analyzed for Th1-type and Th2-type cytokine levels using ELISA. High levels of IFN-γ were detected in group 1 (rL H5-H1N1) and group 2 (rL H5 + IL-2-H1N1). The IFN-γ and IL-10 levels of group 1 (rL H5-H1N1) were significantly higher than in group 3 (H1N1-H1N1) at day 7 p.i. (P < 0.05). The IFN-γ level of group 2 (rL H5 + IL-2-H1N1) was significantly higher than in group 1 (rL H5-H1N1) at day 7 p.i. (P < 0.05). The IFN-γ and IL-10 levels of group 1 and group 2 were significantly elevated after infection as compared to day 0 (P < 0.05) (Figure 2A and D).

The IL-2 and IL-4 levels were not significantly different in all groups of mice (Figure 2B and C). These results suggested that IFN-γ and IL-10 may play roles in HSI. IL-2 co-administrated with rL H5 vaccine could improve the IFN-γ production.

### The percentages of both IFN-γ^+^CD4^+^ T cells and IFN-γ^+^CD8^+^ T cells in spleen

**Figure 3:**
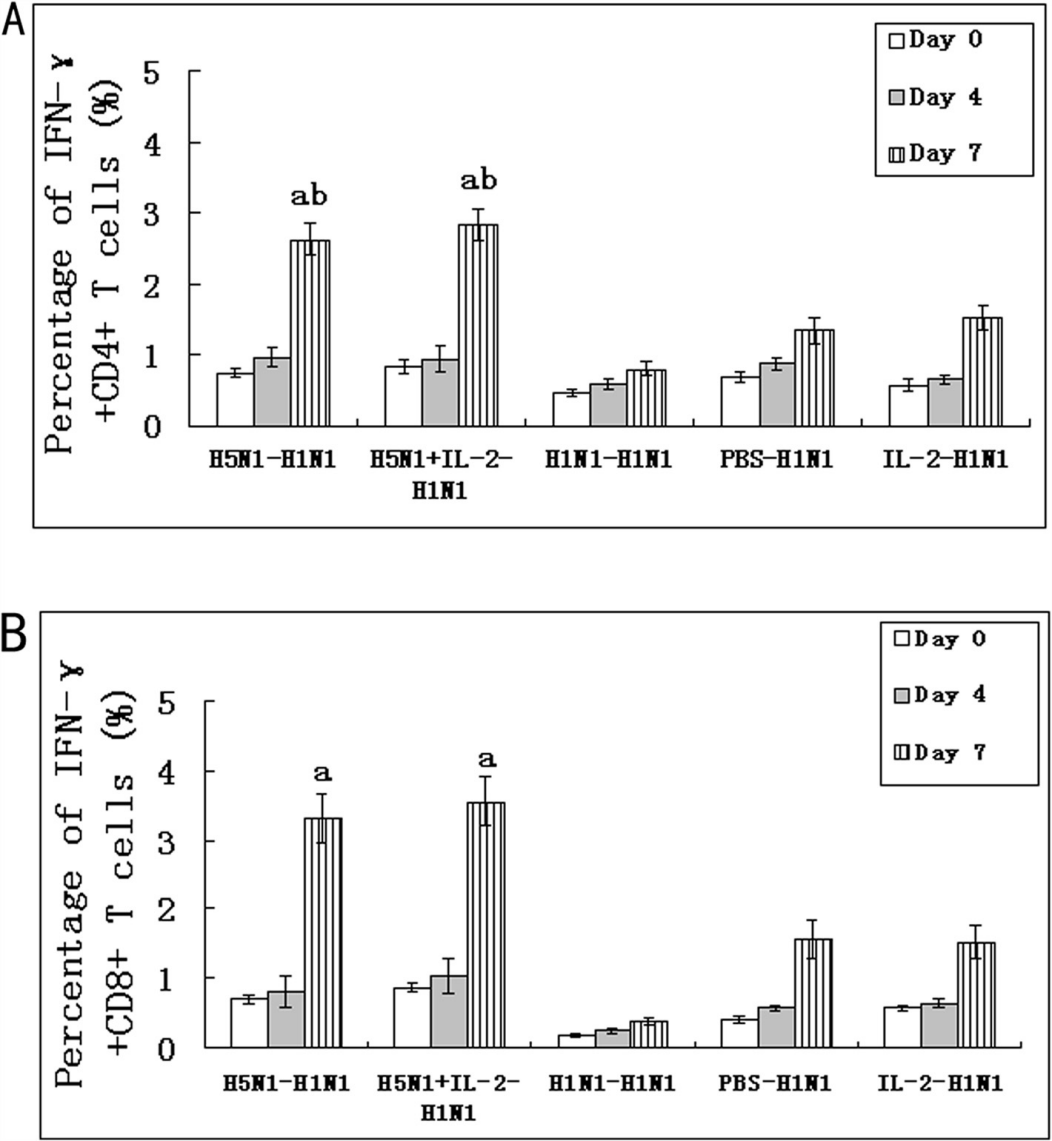
**The percentages of IFN-γ**^**+**^**CD4**^**+ **^**T cells and IFN-γ**^**+**^**CD8**^**+ **^**T cells in spleen.** The percentages of IFN-γ^+^CD4^+^ T cells **(A)** and IFN-γ^+^CD8^+^ T cells **(B)** were assayed using anti-CD3, anti-CD4, anti-CD8b and anti-IFN-γ mAb by flow cytometry. The frequencies of CD3^+^CD4^+^ and CD3^+^CD8^+^ splenocytes were determined by intracellular IFN-γ staining. (**a**, as compare with group 4 (PBS-H1N1), P < 0.05; **b**, as compare with group 3 (H1N1- H1N1), P < 0.05.). n = 3/group.

To investigate the roles of Th1 lymphocytes and cytotoxic T-lymphocytes (CTL) in HSI to influenza virus, the percentages of IFN-γ^+^CD4^+^ and IFN-γ^+^CD8^+^ T cells in spleen were assessed using flow cytometry. The percentages of IFN-γ^+^CD4^+^ and IFN-γ^+^CD8^+^ T cells had not significant change at day 5 p.i.. The percentages of IFN-γ^+^CD4^+^ T cells of mice group 1 (rL H5-H1N1) and group 2 (rL H5 + IL-2-H1N1) were significantly higher than in group 3 (H1N1-H1N1) and group 4 (PBS-H1N1) at day 7 p.i. (P < 0.05) (Figure 3A). The percentages of IFN-γ^+^CD8^+^ T cells of mice group 1 (rL H5-H1N1) and group 2 (rL H5 + IL-2-H1N1) were significantly higher than in group 4 (PBS-H1N1) at day 7 p.i. (P < 0.05) (Figure 3B). These data demonstrated that rL H5 vaccine induced high frequencies of Th1 cells and CTL.

## Discussion

HSI to influenza virus can be successfully induced by live or inactivated whole influenza viruses or DNA vaccines in different kinds of animals [9-13]. In our research, we primed mice with NDV-based recombinant virus vaccine expressing HA gene of influenza A/H5N1 virus(rLH5), and induced cross-protection to influenza A/H1N1 virus infection from severe disease, death and viral replication in the lungs. Ge et al. [14] demonstrated that the NDV portion of rLH5 vaccine protected mice from NDV infection and could not provide protection from influenza A/H5N1 virus infection. We also found that the NDV portion of vaccine does not elicit cross-protection against influenza A/H1N1 virus infection(data not shown). Therefore, the protective efficacy of HSI in our experiments was induced by gene of influenza virus in rLH5 vaccine.

Current vaccines generate neutralizing antibodies directed against viral HA and NA surface glycoproteins of homologous influenza viruses [15]. According to Genbank (ADG59526.1 and AAC53844.1), the amino acid homology of HA of A/Font Monmouth/1/1947 (H1N1) and A/Bar-headed goose/Qinghai/3/2005 (H5N1) was 62%. In this study, antibodies against influenza A/H1N1 virus in sera were determined by HI assay after infection. The results showed a strong antibody response to influenza A/H1N1 virus in group 3 (H1N1-H1N1) that played a critical role in immune responses to homologous influenza virus infection. We observed low or undetectable levels of antibodies to influenza A/H1N1 virus in the other groups. It indicated a negative correlation between HSI and antibody levels.

We found that the IFN-γ and IL-10 levels in the lung samples from group 1 (rL H5-H1N1) and group 2 (rL H5 + IL-2-H1N1) were significantly high after infection in comparison to the other groups . IFN-γ, a Th1 cytokine, can enhance the phagocytic function of macrophages and the cytotoxic effect of NK cells. CTL, which can lyse target cells and prevent spread of influenza virus in vivo, can be activated by IFN-γ [8,16-18]. IL-10 is an anti-inflammatory cytokine. Although IL-10 is a Th2 cytokine, it can inhibit the generation of inflammatory mediators and prevent imbalances of the inflammatory reaction [19]. High levels of IFN-γ and IL-10 can help clear influenza virus, combat excessive inflammation and promote recovery.

T-cell-mediated immune responses are an important factor in HSI to influenza virus. IFN-γ^+^CD4^+^ T cells are Th1 cells and IFN-γ^+^CD8^+^ T cells are effector CTL that are critical features of an adaptive immune system [20-22]. In this study, high frequencies of activated IFN-γ^+^CD4^+^ and IFN-γ^+^CD8^+^ T cells in mice of group 1 (rL H5-H1N1) and group 2 (rL H5 + IL-2-H1N1) were elicited by influenza A/H1N1 virus challenge. It indicated that T-cell-mediated immunity might play an important role in cross-protection between different subtypes of influenza virus.

Vaccines that broadly induce cross-reactive T cell responses are usually directed against conserved viral epitopes of internal proteins, such as NP proteins [23,24]. De Groot et al. [25] reported that the HA also possesses cross-conserved T-cell epitopes [26-28]. We had found that there was no correlation between the levels of antibody and HSI against influenza virus. And our results showed that the levels of IFN-γ and the percentages of both IFN-γ^+^CD4^+^ and IFN-γ^+^CD8^+^ T cells in group 1 (rL H5-H1N1) and group 2 (rL H5 + IL-2-H1N1) increased significantly after infection. We speculate that the cross-protection against heterosubtypic influenza A/H1N1 viruses in mice may be due to cross-conserved T-cell epitope sequences in HA.

Some researchers have used various adjuvants such as IL-2, IL-12, GM-CSF or the heat-labile enterotoxin from enterotoxigenic Escherichia coli to improve immune responses [29,30]. In this study, we selected rIL-2 as an adjuvant to enhance HSI. We found that the mice in the group that co-administered with IL-2 had higher survival rates, lower viral loads and greater amount of IFN-γ production. Henke et al. [31] reported that IL-2 increased the efficacy of DNA immunization to prevent influenza virus infections, IL-2 as an adjuvant for vaccination can stimulate the proliferation and activation of T cells, NK cells and macrophage. It can also enhance cellular immunity [32]. Further studies will be done to clarify the mechanism that IL-2, as an adjuvant, enhance HSI.

In summary, rLH5 vaccine induced cross-protective immune response against heterosubtypic influenza A/H1N1 virus infection in mice, and this cross-protection was improved with IL-2 as an adjuvant. T cell immunity may play an important role in HSI against influenza virus. Further studies will be necessary to determine the most effective cross-conserved T-cell epitope to HSI and the precise mechanism of HSI.

## Materials and Methods

### Mice

Healthy female C57BL/6 mice 6–8 weeks of age were obtained from the Laboratory Animal Center of China Medical University. Mice were bred and/or housed under specific pathogen-free (SPF) conditions until the day of sacrifice. The Institutional Ethics Committee for Animal Use and Care approved all animal-related experiments and procedures.

### Influenza virus and vaccine

Influenza viruses A/Font Monmouth/1/1947 (A/FM/1/47) (H1N1) were propagated in the allantoic cavity of 10 day-old embryonated SPF chicken eggs at 34°C, and allantoic fluid was harvested after 2–3 days. Fifty percent egg infectious dose (EID_50_) titers were determined by serial titration of viruses in eggs and calculated by the method of Reed and Muench [8].

rL H5 vaccine was purchased from Harbin Veterinary Research Institute. HA open reading frame from A/Bar-headed goose/Qinghai/3/2005 (H5N1) were inserted into the intergenic region between the P and M genes of the LaSota NDV vaccine strain.

### Immunization and challenge of mice

Mice were anesthetized by intraperitoneal injection with tribromoethanol (Avertin; 250 to 300 μl per mouse) [33]. Mice were intranasally inoculated with 10^6.0^ EID_50_ of rL H5 vaccine in a volume of 50 μl. Three weeks later, the mice were re-immunized in the same manner with the same amount of the vaccine. Two weeks after re-immunization, mice were intranasally infected with 10^4.8^ EID_50_ of influenza A/H1N1 virus in a volume of 50 μl. Mice were divided into six groups (Table 2). Group 1 (rL H5-H1N1): Mice were primed with rL H5 vaccine and infected with influenza A/H1N1 virus as described above; Group 2 (rL H5 + IL-2-H1N1): Mice were primed with rL H5 vaccine and 160 U of rIL-2 in a volume of 100 μl PBS was co-administered intraperitoneally 3 days after the primary and secondary immunization. Mice were challenged with influenza A/H1N1 virus as in group 1; Group 3 (H1N1-H1N1): Mice were intranasally inoculated with 10^2.8^ EID_50_ of influenza A/H1N1 virus in a volume of 50 μl and were challenged with influenza A/H1N1 virus as in group 1 [20]; Group 4 (PBS-H1N1): Mice were given PBS in a volume of 50 μl and were challenged with influenza A/H1N1 virus as in group 1; Group 5 (IL-2-H1N1): Mice were injected intraperitoneally with rIL-2 in the same manner as group 2 and were challenged with influenza A/H1N1 virus as in group 1; Group 6: Negative control group.

**Table 2:** Experimental groups

**Experimental group**	**Vaccination**			**H1N1 challenge**
	**Recombinant virus vaccine**	**Adjuvant**	**H1N1**	
**Group 1 (rLH5-H1N1)**	**+**	**-**	**-**	**+**
**Group 2 (rLH5 + IL-2-H1N1)**	**+**	**+**	**-**	**+**
**Group 3 (H1N1-H1N1)**	**-**	**-**	**+**	**+**
**Group 4 (PBS-H1N1)**	**-**	**-**	**-**	**+**
**Group 5 (IL-2-H1N1)**	**-**	**+**	**-**	**+**
**Group 6**	**-**	**-**	**-**	**-**

Mice (n = 12/group) were monitored daily for body weight and survival rates after infection. At day 0, 5 and 7 p.i., mice were euthanized and their lungs and spleen were resected.

### Lung index assays

Three mice from each group were euthanized and their lungs were weighed. Lung index is expressed as percentage of weight, according to the formula: *lung weight/body weight × 100%*.

### HI assay

Mice (n = 3) from each group were bled via the orbital sinus vein and the sera were collected at day 7 and 14 p.i.. HI assay was performed as previously described [34,35].

### Cytokine assays

Intact lungs of mice (n = 3) from each group were prepared to assess cytokine levels at days 0 and 7 p.i.. Whole lungs were homogenized using a conventional method [36]. Homogenates were centrifuged at 300 g for 10 min., and supernatants were collected. The amounts of interferon-γ (IFN-γ), IL-2, IL-4 and IL-10 in lung suspensions were measured using ELISA as described previously [34].

### Flow-cytometric analysis

The virus-specific IFN-γ^+^CD4^+^ and IFN-γ^+^CD8^+^ T cells were analyzed using flow cytometry. Splenocytes of mice (n = 3) from each group were collected at day 0, 5 and 7 p.i.. Single-cell suspensions were prepared as previously described [9]. 1 × 10^6^ splenocytes were plated to a 96-well flat-bottom plate and stimulated with infectious influenza A/H1N1 virus at an MOI of 0.1 in 200 μl volume for 21 h [27]. Bredfeldin A (BD GolgiPlug) was added at a final concentration of 1 μg/ml for the last 5 h of incubation to block protein transport. Cells were stained intracellularly for fluorescein isothiocyanate (FITC)-anti-INF-γ after surface staining for Peridinin-Chlorophyll-Protein Complex (PerCP)-anti-CD3, phycoerythrin (PE)-anti-CD4 or PE-anti-CD8b (Becton Dickinson). Stained cells were quantified using a FACS Calibur (BD Biosciences) and analysed using the FlowJo software (Treestar).

### Virus quantification assays

Lung suspensions of mice (n = 3) from each group were prepared at day 5 p.i.. Influenza virus in the lung suspensions was quantified using real-time RT-PCR [37]. Total virus RNA was prepared using the RNAiso Plus (TaKaRa) following the manufacturer’s instructions. The primer sequences used for RT-PCR and quantitative real time PCR of the viral NP gene were 5′-CTGAGAAGCAGGTACTGGGC-3′ (sense) and 5′-CTGCATTGTCTCCGAAGAAAT-3′ (antisense).

Real-time RT-PCR was performed using the SYBR^®^ PrimeScript^®^ RT-PCR kit (Perfect Real Time, TaKaRa). A 20 μl reaction volume was prepared according to the manufacturer’s instructions. Cycling conditions for real-time RT-PCR were as follows: 37°C for 15 min, 85°C for 5 s, 95°C for 30 s followed by 40 cycles of 95°C for 5 s, 55°C for 34 s. Real-time RT-PCR was conducted using the ABI 7500 and the data were analyzed using ABI software.

Conventional RT-PCR was carried out using the extracted RNA and primers as described above. The concentration of the RT-PCR product was determined by measuring the OD at 260 nm using a spectrophotometer (Eppendorf) [38]. Serial 10-fold dilutions of the product DNA with EASY dilution (TaKaRa) were used in real-time PCR and the standard curve generated from the amplification plot.

### Statistical analysis

Statistical analyses were carried out using the SPSS 17.0 software. The differences between values were evaluated through a one-way analysis of variance (ANOVA) followed by pair-wise comparison with the Student-Newman-Keuls test. P < 0.05 was considered statistically significant and all values are expressed as means ± SEM. All experiments were performed at least three times.

## Abbreviations

HIS: Heterosubtypic immunity; rL H5: Recombinant virus vaccine; HA: Hemagglutinin; NA: Neuraminidase; NDV: Newcastle disease virus; HI: Hemagglutination inhibition; IAV: Influenza A virus; rIL-2: Interleukin-2; SPF: Specific pathogen-free; EID50: Fifty percent egg infectious dose; PBS: Phosphate-buffered saline; p.i.: Post-infection; IFN-γ: Interferon-γ; CTL: Cytotoxic T-lymphocytes.

## Competing interests

None of the authors has a financial or personal competing interest related to this study.

## Authors’ contributions

SY proposed the idea, analyzed and interpreted the data and wrote the manuscript, HJ revised the manuscript critically for important intellectual content and study design. YY, KX and SL participated in data analysis and interpretation of data. SN and ZG revised the manuscript. SY and SN administrative, technical, or materiel support and revised the manuscript. All authors read and approved the final manuscript.

## References

[B1] Tong SX Li Y Rivailler P Conrardy C Castillo DA Chen LM Recuenco S Ellison JA Davis CT York IA Turmelle AS Moran D Rogers S Shi M Tao Y Weil MR Tang K Rowe LA Sammons S Xu X Frace M Lindblade KA Cox NJ Anderson LJ Rupprecht CE Donis RO A distinct lineage of influenza A virus from bats Proc Natl Acad Sci U S A 2012 10 4269 4274 22371588 10.1073/pnas.1116200109 PMC3306675

[B2] Zhu XY Yu WL McBride R Li Y Chen LM Donis RO Tong S Paulson JC Wilson IA Hemagglutinin homologue from H17N10 bat influenza virus exhibits divergent receptor-binding and pH-dependent fusion activities Proc Natl Acad Sci U S A 2013 10 1458 1463 10.1073/pnas.1218509110 23297216 PMC3557073

[B3] He W Han H Wang W Gao B Anti-influenza virus effect of aqueous extracts from dandelion J Virol 2011 10 538 558 10.1186/1743-422X-8-538 PMC3265450 22168277

[B4] Manicassamy B Medina RA Hai R Tsibane T Stertz S Nistal-Villán E Palese P Basler CF García-Sastre A Protection of mice against lethal challenge with 2009 H1N1 influenza A virus by 1918-like and classical Swine H1N1 based vaccines PLoS Pathog 2010 10 http://dx.doi.org/10.1371/journal.ppat.1000745 20126449 10.1371/journal.ppat.1000745 PMC2813279

[B5] Palese P Shaw ML Howley DMKM Orthomyxoviridae: the viruses and their replication Fields virology 2007 5 1647 1689

[B6] Bodewes R Kreijtz JH Geelhoed-Mieras MM van Amerongen G Verburgh RJ van Trierum SE Kuiken T Fouchier RA Osterhaus AD Rimmelzwaan GF Vaccination against seasonal influenza A/H3N2 virus reduces the induction of heterosubtypic immunity against influenza A/H5N1 virus infection in ferrets J Virol 2011 10 2695 2702 10.1128/JVI.02371-10 21228239 PMC3067975

[B7] Lu X Edwards LE Desheva JA Nguyen DC Rekstin A Stephenson I Szretter K Cox NJ Rudenko LG Klimov A Katz JM Cross-protective immunity in mice induced by live-attenuated or inactivated vaccines against highly pathogenic influenza A(H5N1) viruses Vaccine 2006 10 6588 6593 10.1016/j.vaccine.2006.05.039 17030078

[B8] Seo SH Peiris M Webster RG Proective cross-reactive cellular immunity to lethal A/Goose/Guangdong/1/96-like H5N1 influenza virus CD8+ T cell expressing gamma interferon J Virol 2002 10 4886 4890 10.1128/JVI.76.10.4886-4890.2002 11967305 PMC136145

[B9] Kreijtz JH Bodewes R van den Brand JM de Mutsert G Baas C van Amerongen G Fouchier RA Osterhaus AD Rimmelzwaan GF Infection of mice with a human influenza A/H3N2 virus induces protective immunity against lethal infection with influenza A/H5N1 virus Vaccine 2009 10 4983 4989 10.1016/j.vaccine.2009.05.079 19538996

[B10] Chen Q Madson D Miller CL Harris DL Vaccine development for protecting swine against influenza virus Anim Health Res Rev 2012 10 181 195 10.1017/S1466252312000175 23253165

[B11] Hikono H Mase M Matsuu A Nakayama M Saito T Intraocular vaccination with an inactivated highly pathogenic avian influenza virus induces protective antibody responses in chickens Vet Immunol Immunopathol 2013 10 83 89 10.1016/j.vetimm.2012.10.005 23159237

[B12] Huang B Wang W Li R Wang X Jiang T Qi X Gao Y Tan W Ruan L Influenza A virus nucleoprotein derived from Escherichia coli or recombinant vaccinia (Tiantan) virus elicits robust cross-protection in mice J Virol 2012 10 322 334 10.1186/1743-422X-9-322 PMC3547759 23272943

[B13] Prabakaran M Kolpe AB He F Kwang J Cross-protective efficacy of bivalent recombinant baculoviral vaccine against heterologous influenza H5N1 challenge Vaccine 2013 10 1385 1392 10.1016/j.vaccine.2013.01.003 23328313

[B14] Ge J Deng G Wen Z Tian G Wang Y Shi J Wang X Li Y Hu S Jiang Y Yang C Yu K Bu Z Chen H Newcastle disease virus-based live attenuated vaccine completely protects chickens and mice from lethal challenge of homologous and heterologous H5N1 avian influenza viruses J Virol 2007 10 150 158 10.1128/JVI.01514-06 17050610 PMC1797253

[B15] Teijaro JR Verhoeven D Page CA Turner D Farber DL Memory CD4 T cells direct protective responses to influenza virus in the lungs through helper-independent mechanisms J Virol 2010 10 9217 9220 20592069 10.1128/JVI.01069-10 PMC2937635

[B16] Rimmelzwaan GF Boon AC Geelhoed MM Voeten JT Fouchier RA Osterhaus AD Human airway epithelial cells present antigen to influenza virus-specific CD8+ CTL inefficiently after incubation with viral protein together with ISCOMATRIX Vaccine 2004 10 2769 2775 15246610 10.1016/j.vaccine.2004.01.052

[B17] Veckman V Miettinen M Pirhonen J Siren J Matikainen S Julkunen I Streptococcus pyogense and lactobacillus rhamnosus differentially induce maturation and production of Th1-type cytokines and chemokines in human monocyte-derived dendritic cells J Leukoc Biol 2004 10 764 771 14966192 10.1189/jlb.1003461

[B18] Osterlund P Pirhonen J Ikonen N Ronkko E Strengell M Makela SM Broman M Hamming OJ Hartmann R Ziegler T Julkunen I Pandemic H1N1 2009 influenza A virus induces weak cytokine responses in human macrophages and dendritic cells and is highly sensitive to the antiviral actions of interferons J Virol 2010 10 1414 1422 10.1128/JVI.01619-09 19939920 PMC2812319

[B19] Seitz MLP Dewald B Towbln H Towbin H Gallati H Baggiolini M IL-10 differentially regulates cytokine inhibitor and chemokine release from blood mononuclear cells and fibroblasts Eur J Immunol 1995 10 1129 1132 10.1002/eji.1830250443 7737285

[B20] Epstein SL Lo CY Misplon JA Bennink JR Mechanism of protective immunity against influenza virus infection in mice without antibodies J Immunol 1998 10 322 327 9551987

[B21] Ge X Tan V Bollyky PL Standifer NE James EA Kwok WW Assessment of seasonal influenza A virus-specific CD4 T- cell resonses to 2009 pandemic H1N1 Swine-Origin influenza A virus J Virol 2010 10 3312 3319 10.1128/JVI.02226-09 20071564 PMC2838145

[B22] Grebe KM Yewdell JW Bennink JR Heterosubtypic immunity to influenz A virus: where do we stand? Microbes Infect 2008 10 1024 1029 10.1016/j.micinf.2008.07.002 18662798 PMC2584237

[B23] Kreijtz JH de Mutsert G van Baalen CA Fouchier RA Osterhaus AD Rimmelzwaan GF Cross-recognition of avian H5N1 influenza virus by human cytotoxic T-lymphocyte populations directed to human influenza A virus J virology 2008 10 5161 5166 10.1128/JVI.02694-07 18353950 PMC2395172

[B24] Tu W Mao H Zheng J Liu Y Chiu SS Qin G Chan PL Lam KT Guan J Zhang L Guan Y Yuen KY Peiris JS Lau YL Cytotoxic T lymphocytes established by seasonal human influenza cross-react against 2009 pandemic H1N1 influenza virus J virol 2010 10 6527 6535 10.1128/JVI.00519-10 20410263 PMC2903266

[B25] De Groot AS Ardito M McClaine EM Moise L Martin WD Immunoinformatic comparison of T-cell epitopes contained in novel swine-origin influenza A (H1N1) virus with epitopes in 2008–2009 conventional influenza vaccine Vaccine 2009 10 5740 5747 10.1016/j.vaccine.2009.07.040 19660593

[B26] Hessel A Schwendinger M Holzer GW Orlinger KK Coulibaly S Savidis-Dacho H Zips ML Crowe BA Kreil TR Ehrlich HJ Barrett PN Falkner FG Vectors based on modified vaccinia Ankara expressing influenza H5N1 hemagglutinin induce substantial cross-clade protective immunity PLoS One 2011 10 http://dx.doi.org/10.1371/journal.pone.0016247 21283631 10.1371/journal.pone.0016247 PMC3026016

[B27] Brewoo JN Powell TD Jones JC Gundlach NA Young GR Chu H Das SC Partidos CD Stinchcomb DT Osorio JE Cross-protective immunity against multiple influenza virus subtypes by a novel modified vaccinia Ankara (MVA) vectored vaccine in mice Vaccine 2013 10 1848 1855 10.1016/j.vaccine.2013.01.038 23376279 PMC4224110

[B28] Bui HH Peters B Assarsson E Mbawuike I Sette A Ab and T cell epitopes of influenza A virus, knowledge and opportunities Proc Natl Acad Sci U S A 2007 10 1 246 251 10.1073/pnas.0609330104 17200302 PMC1765443

[B29] Slpushkin VA Katz JM Black RA Gamble WC Rota PA Cox NJ Protection of mice against influenza A virus challenge by vaccination with baculovius-expressed Mz protein Vaccine 1995 10 1399 1402 10.1016/0264-410X(95)92777-Y 8578816

[B30] Katz JM Lu X Young SA Galphin JC Aduvant activity of the heat-labile enterotoxin from enterotoxigenic Escherichia coli for oral administration of inactivated influenza virus vaccine J Infect Dis 1997 10 352 363 10.1093/infdis/175.2.352 9203656

[B31] Henke A Rohand N Zell R Wutzler P Co-expression of interleukin-2 by a bicistronic plasmid increases the efficacy of DNA immunization to prevent influenza virus infections Intervirology 2006 10 249 252 10.1159/000092487 16601357

[B32] Chow YH Huang WL Chi WK Chu YD Tao MH Improvement of hepatitis B virus DNA vaccines by plasmids coexpressing hepatitis B surface antigen and interleukin-2 J Virol 1997 10 169 178 8985336 10.1128/jvi.71.1.169-178.1997 PMC191037

[B33] Richards KA Chaves FA Sant AJ Infection of hla-dr1 transgenic mice with a human isolate of influenza A virus (H1N1) primes s diverse CD4 T-cell repertoire that includes CD4 T cell with heterosubtypic cross-reactivity to avian(H5N1) influenza virus J Virol 2009 10 6566 6577 10.1128/JVI.00302-09 19386707 PMC2698557

[B34] Sambhara S Kurichhh A Miranda R Tumpey T Rowe T Renshaw M Arpino R Tamane A Kandil A James O Underdown B Klein M Katz J Burt D Haterosubtypic immunity against human influenza A viruses, indcluding recently emerged avian H5 and H9 viruses, induced by FLU-ISCOM vaccine in mice requires both cytotoxic T-lymhocyte and macrophage function Cell Immunol 2001 10 143 153 10.1006/cimm.2001.1835 11591118

[B35] Rowe T Abernathy RA Hu-Primmer J Thompson WW Lu X Lim W Fukuda K Cox NJ Katz JM Detection of antibody to avian influenza A (H5N1) virus in human serum by using a combination of serologic assays J Clin Microbiol 1999 10 937 943 10074505 10.1128/jcm.37.4.937-943.1999 PMC88628

[B36] Nguyen HH van Ginkel FW Vu HL McGhee JR Mestecky J Heterosubtypic immunity to influenza A virus infection requires B cells but not CD8+ cytotoxic T lymphocytes J Infect Dis 2001 10 368 376 10.1086/318084 11133367

[B37] CDC CDC Protocol for realtime RT PCR for influenza A (H1N1)-revision 2009

[B38] Wan C Huang Y Cheng L Fu G Shi S Chen H Peng C Lin F Lin J The development of a rapid SYBR Green I-based quantitative PCR for detection of duck circovirus J Virol 2011 10 465 470 10.1186/1743-422X-8-465 PMC3198713 21978576

